# Investigating the clinical potential for 14-3-3 zeta protein to serve as a biomarker for epithelial ovarian cancer

**DOI:** 10.1186/1757-2215-6-79

**Published:** 2013-11-15

**Authors:** Ioannis Hatzipetros, Peter Gocze, Tamas Koszegi, Akos Jaray, Laszlo Szereday, Beata Polgar, Nelli Farkas, Balint Farkas

**Affiliations:** 1 Department of Obstetrics and Gynecology, University of Pecs, Clinical Center, Edesanyak Str. 17, 7624 Pecs, Hungary; 2 Institute of Laboratory Medicine, University of Pecs, Hungary; 3 Department of Radiology, University of Pecs, Hungary; 4 Department of Medical Microbiology and Immunology, University of Pecs, Hungary; 5 Institute of Bioanalysis, University of Pecs, Hungary

**Keywords:** Epithelial ovarian cancer, 14-3-3 Zeta protein, CA-125, HE-4, Chemotherapy, ROMA

## Abstract

**Objective:**

Recently, 14-3-3 zeta protein was identified as a potential serum biomarker of epithelial ovarian cancer (EOC). The goal of this study was to investigate the clinical potential of 14-3-3 zeta protein for monitoring EOC progression compared with CA-125 and HE4.

**Design:**

Prospective follow-up study.

**Setting:**

University of Pecs Medical Center Department of Obstetrics and Gynecology/Oncology (Pecs, Hungary).

**Population:**

Thirteen EOC patients with advanced stage (FIGO IIb-IIIc) epithelial ovarian cancer that underwent radical surgery and received six consecutive cycles of first line chemotherapy (paclitaxel, carboplatin) in 21-day intervals.

**Methods:**

Pre- and post-chemotherapy computed tomography (CT) scans were performed. Serum levels of CA-125, HE4, and 14-3-3 zeta protein were detected by enzyme-linked immunosorbent assay (ELISA) and quantitative electrochemiluminescence assay (ECLIA).

**Main outcome measures:**

Serum levels of CA-125, HE4, and 14-3-3 zeta protein, as well as lesion size according to pre- and post-chemotherapy CT scans.

**Results:**

Serum levels of CA-125 and HE4 were found to significantly decrease following chemotherapy, and this was consistent with the decrease in lesion size detected post-chemotherapy. In contrast, 14-3-3 zeta protein levels did not significantly differ in healthy postmenopausal patients versus EOC patients.

**Conclusions:**

Determination of CA-125 and HE4 serum levels for the determination of the risk of ovarian malignancy algorithm (ROMA) represents a useful tool for the prediction of chemotherapy efficacy for EOC patients. However, levels of 14-3-3 zeta protein were not found to vary significantly as a consequence of treatment. Therefore we question if 14-3-3 zeta protein is a reliable biomarker, which correlates with the clinical behavior of EOC.

## Introduction

Epithelial ovarian cancer (EOC) is a highly malignant gynecological neoplasia with an incidence of 12/100 000 women [1], and this rate has only slightly decreased in the last 80 years. While women of any age are at risk for this malignancy, postmenopausal women have a higher incidence. For example, 90% of women who suffer from EOC are older than 40 years of age, and the greatest number are 55 years or older. Moreover, due to the anatomic position of the ovaries, pelvic malignancies can remain obscured. In addition, a lack of symptoms until the advanced stages of tumor development results in an increased rate of metastasis at the time of diagnosis. Correspondingly, 80% of the ovarian neoplasias diagnosed are stage III-IV according to International Federation of Obstetrics and Gynecology (FIGO) criteria. While FIGO stage I EOC has a relatively high five-year survival rate (> 90%), survival rates markedly drop for patients with stage III-IV EOC (25–30%) [2,3]. Therefore, it is crucial to diagnose EOC as early as possible. Accordingly, the identification of serum biomarkers to detect EOC would represent an important and valuable advance for the monitoring and treatment of EOC progression.

Algorithms and triage protocols designed to evaluate potential cases of ovarian cancer in their early stages are currently limited, and rely on pelvic sonography and CA-125 determination [4]. Moreover, the sensitivity and specificity of these approaches range from 70–80%. [5]. Regarding CA-125, its levels are elevated in less than 50% of EOC cases, and it is undetectable in another 20% of EOC cases. In addition, high serum levels of CA-125 are also associated with benign gynecological diseases (e.g., cysts, endometriosis, etc.) [6]. In 2008, Moore and colleagues identified human epididymis protein 4 (HE4) as a biomarker for ovarian cancer [7]. Based on these findings, a risk of ovarian malignancy algorithm (ROMA) was developed, and is currently used to predict the presence of malignant ovarian cancer using a combination of CA-125 levels, HE4 expression, and menopausal status. In particular, the combination of HE4 and CA-125 in the ROMA has been associated with a higher sensitivity than any single biomarker [8].

14-3-3 zeta is an important regulatory protein, which mediates intracellular signaling pathways by interfering with approximately 100 cellular proteins, including oncogenes and protooncogenes. Recently, two independent research groups, Waldemarson et al. [9] and He et al*.*[10], advocated 14-3-3 zeta as a potential biomarker for EOC. In addition, Kobayashi et al. [11] recently demonstrated that 14-3-3 zeta protein is present in malignant ascites of patients with EOC, and is secreted by ascetic monocytes and macrophage. However, while the role of 14-3-3 zeta protein as an intracellular adaptor protein has been widely investigated, the function of the secreted protein is unclear. Therefore, the goal of the current pilot study was to assess the potential for 14-3-3 zeta protein to serve as a biomarker for monitoring patients with FIGO stage II-III EOC that undergo chemotherapy.

## Materials and methods

### Patients and follow-up study design

This prospective study was approved by the University of Pecs Institutional Ethical Review Board (#4076.316-251/KK15/2011), and written informed consent was obtained from all enrolled patients.

Peripheral blood samples were collected preoperatively from 13 patients admitted for six cycles of first line chemotherapy (paclitaxel/carboplatin; Hungarian OEP Chemotherapy protocol # 7167). Chemotherapy dosage was calculated based on body mass (kg), and treatments were administered in 21 d intervals at the University of Pecs Medical Center Department of Obstetrics and Gynecology/Oncology (Pecs, Hungary) in 2012. Blood samples were collected 1–2 h before each treatment into citrate tubes. These tubes were then centrifuged (5000 rpm for 10 min), and blood plasma samples were collected and stored at −80°C. When needed, samples were thawed at room temperature (RT), and then were thoroughly vortexed as indicated by the manufacturer’s recommendation. All patients, aged 41 to 73 y (mean, 60 y), underwent radical gynecological surgery for the removal of both adnexes with or without the uterus. However, peritoneal or lymph node metastases were not resected. Computed tomography (CT) scans were performed prior to and following the completion of chemotherapy treatment. These images were used to assess changes in both target and non-target lesions. Each diagnosis was verified according to histopathology studies of the original tumors. Histopathological grade and stage of disease (according to FIGO criteria) were available for all malignant cases, and included FIGO stage IIa (n = 2), stage IIIb (n = 2), and stage IIIc (n = 9) cases (Table 1).

**Table 1:** Clinicopathological features of the patients enrolled in this study that underwent six cycles of paclitaxel/carboplatin-based chemotherapy for treatment of EOC

**Patient no.**	**Histology**	**FIGO stage**	**Age, y**	Tumor grade*
1	Serous	III C	69	High
2	Serous	III C	57	High
3	Serous	III B	41	High
4	Serous	II A	50	High
5	Serous	III C	67	High
6	Serous	III C	64	High
7	Serous	III B	73	High
8	Adenosquamous	II B	51	High
9	Adenosquamous	III C	69	High
10	Serous	III C	55	High
11	Serous	III C	71	High
12	Serous	III C	60	High
13	Serous	III C	53	High

*According to International Federation of Obstetrics and Gynecology (FIGO) criteria.

### Computed tomography (CT) scans

CT scans were performed in the Department of Radiology (University of Pecs, Hungary). Contiguous 5 mm axial slices obtained through the abdomen and pelvis. Prior to examination high-concentration iodinated contrast agent was administered intravenously (Iomeron 400, Bracco Diagnostic Imaging). Field-of-view was adjusted to body habitus (to include the whole body including the skin). Target and non-target lesions were defined based on RECIST 1.1 guidelines (http://www.recist.com). A lesion was measurable and defined as a target lesion if the tumor was ≥ 10 mm along its longest diameter (LD) on a CT axial image with ≤ 5 mm reconstruction intervals, or if lymph nodes were ≥ 15 mm along their short axis on CT images. Non-target lesions were considered to be: masses with a diameter < 10 mm, lymph nodes with a diameter of 10–14 mm along the short axis, ascites, pleural or pericardial effusion, abdominal masses, or organomegaly identified by physical exam. Furthermore, these could not be measured by reproducible imaging techniques. CT scans were performed 1–2 weeks after radical surgeries were performed, 1–2 weeks prior to chemotherapy, and 1–2 weeks after the final chemotherapy treatment.

### Laboratory methods

#### Enzyme-linked immunosorbent assay (ELISA)

Levels of serum CA-125 (Fujirebio Diagnostics, Malvern, PA; Catalog #: 400–10, Lot. # 29191), HE4 (Fujirebio Diagnostics, Malvern, PA; Catalog #: 404–10, Lot# 28373), and 14-3-3 zeta protein (Cusabio Biotech, Wuhan, China; Catalog #: CSB-EL026293HU, Lot. #A26174460) were determined using a quantitative sandwich enzyme immunoassay according to each manufacturer’s protocol. Serum concentrations were calculated using Optima 2.10 R2 built-in data calculator software.

### Quantitative electrochemiluminescence assay (ECLIA)

Tumor marker levels were measured using a Roche electrochemiluminescent fully automated immunoassay system (ECLIA, Roche Diagnostics, http://www.roche-diagnostics.com). To determine serum levels of CA-125 (Cat. no. 11776223), and HE4 (Cat. no. 05950929), samples were processed using a Roche Cobas e411 analyzer. Master calibration, imprecision, and inaccuracy were checked using bi-level quality controls prior to the analysis of patient serum samples.

### Risk for ovarian malignancy algorithm (ROMA) index

The ROMA used serum levels of HE4 and CA-125 measured either by ELISA or ECLIA, and was calculated using an Excel spreadsheet with preset formulas to generate the predictive index (PI) value for EOC [12] as follows:

For postmenopausal women: PI = −8.09 + 1.04*LN [HE4] + 0.732*LN [CA125].

A ROMA value was then calculated as follows: ROMA value (%) = exp(PI) / [1 + exp(PI)]*100. According to the manufacturer’s manual, the detection of HE4 by ECLIA and CA-125 by ELISA in menopausal women identified an EOC high-risk index value equal to, or higher than, 25.3% [13].

### Statistical analysis

Statistical analyses were performed using IBM SPSS Statistic 20 (IBM Corporation) at the University of Pecs, Institute of Bioanalysis. The sample size (n) was 13, and comparisons were made between treatments and between methods according to the Wilcoxon signed-rank test. To evaluate trends between the number of treatments and serum levels of tumor markers, linear regression and correlation analyses were applied. To examine the relationship between tumor marker levels and CT scan results, Spearman’s rank correlation coefficient was used. Mean data are reported ± standard error of the mean (SEM). Statistical significance was set at *p* < 0.05, or *p* < 0.1.

## Results

### Radiologic assessment following therapeutic procedures

**Figure 1:**
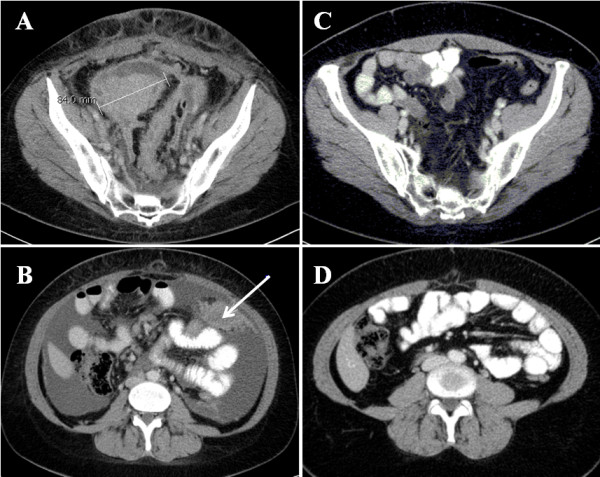
**Axial CT slices of patient # 3 after contrast material was intravenously administered.** A target lesion with a SLD of 84 mm is localized near the minor pelvis before **(A)** and after **(C)** six cycles of first-line chemotherapy. **(B)** The arrow represents a mesenteric peritoneal carcinosis (non-target lesion) in the same patient that is level with the lower edge of the liver prior to chemotherapy. A significant amount of ascites associated with the non-target lesion is also observed **(B)**. **(D)** Both non-target lesions are absent after the completion of chemotherapy.

CT scans were obtained one to two weeks after radical gynecological surgeries were performed. After an initial laparotomy, 10/13 (76.92%) patients were found to have residual tumor present prior to induction of paclitaxel/carboplatin-based chemotherapy. After six consecutive cycles of treatment within 21 d intervals, CT scans were repeated. At this point, residual tumor with a LD value >1 cm was only detected in 4/13 (30.76%) patients (Table 2). Based on the detection of 26 non-target lesions in pre-chemotherapy CT scans, and only 3 non-target lesions in post-chemotherapy scans, the efficacy of chemotherapy for EOC treatment is demonstrated (Figure 1).

**Table 2:** Evaluation of tumor size before and after paclitaxel/carboplatin chemotherapy using CT scans

**Patient no.**	**SLD of target lesions 10 (+/− 4) d before chemotherapy (mm)**	**SLD of target lesions 10 (+/− 4) d after the last cycle of chemotherapy (mm)**	Non-target lesion changes*
1	22	0	−1
2	56	0	−1
3	84	0	−1
4	0	0	0
5	137	0	−4
6	0	0	0
7	0	0	0
8	441	202	−1
9	256	18	−3
10	147	0	−3
11	125	0	−1
12	46	18	−1
13	39	14	−3

SLD: sum of length diameter; according to RECIST 1.1 guidelines.

*Negative values represent a decrease in lesion size.

### Detection of CA-125, HE4, 14-3-3 zeta protein

**Figure 2:**
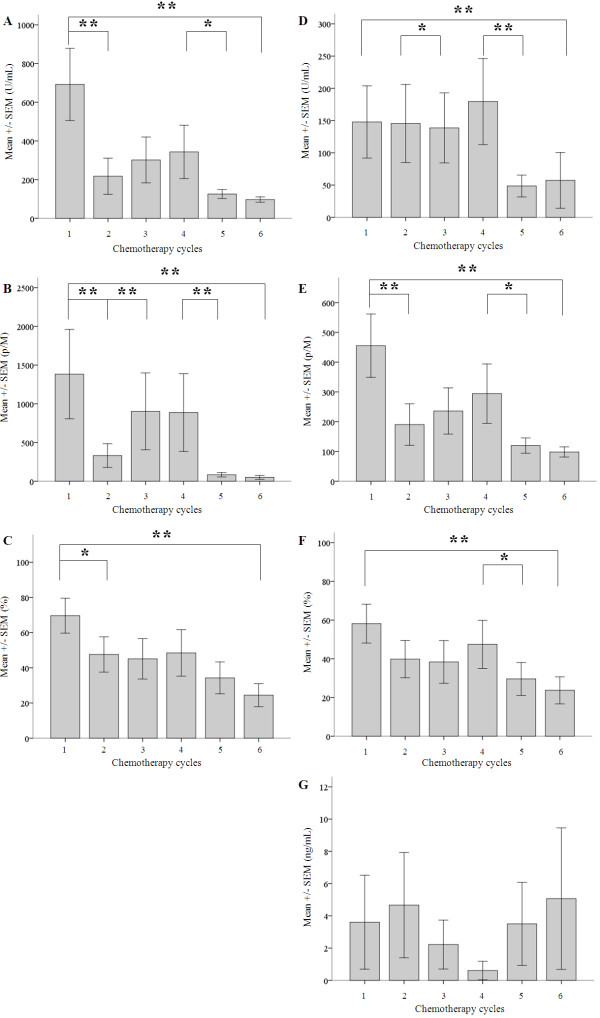
**Mean levels of serum biomarkers (CA-125, HE4 and 14-3-3 zeta protein) measured with ELISA and ECLIA methods, and the ROMA index values.** Average values of CA-125 (U/mL) +/− SEM determined by ECLIA **(A)** and by ELISA **(D)** are shown according to the chemotherapy cycles (consistently on each x-axis from 1 to 6). Mean concentrations of HE4 (p/M), measured by ECLIA **(B)** and by ELISA **(E)** are also shown. Furthermore average values determined by ELISA **(G)** of 14-3-3 zeta protein (ng/ml) are depicted. Based on the above-mentioned data, we calculated the postmenopausal ROMA index values (%) for ECLIA **(C)** and for ELISA **(F)** techniques. We represent the mean levels of 14-3-3 zeta protein (ng/mL) for each of the treatment days. Wilcoxon signed-rank test analysis was performed for each diagram, and statistical significance represented with asterisk (*) was set at p < 0.05, and (**) when p < 0.01.

Levels of CA-125, HE4, and 14-3-3 zeta protein were monitored throughout the treatment period by ELISA and ECLIA. On the first day of chemotherapy, the mean concentration of CA-125 was 147.87 ± 55.98 U/ml and 648.26 ± 186.52 U/ml, respectively. After completing the sixth cycle of chemotherapy, CA-125 levels were lower, with the mean concentrations detected being 58.54 ± 30.89 U/ml and 119.70 ± 22.75 U/ml, respectively. Similarly, mean serum levels of HE4 detected on the first day of chemotherapy by ELISA were 455.32 ± 106.39 pM, and decreased to 120.52 ± 23.76 pM upon completion of chemotherapy. Using the ECLIA method, serum levels of HE4 were 1383.49 ± 577.23 pM on the first day of chemotherapy, and decreased to 70.12 ± 26.44 pM upon completion of chemotherapy. ROMA index values were subsequently calculated, and decreased from 58.17 ± 10.05% to 28.95 ± 7.67%, and from 69.62 ± 9.91% to 30.78 ± 7.91% for ELISA and ECLIA, respectively. According to Wilcoxon statistical analyses, the differences in the values determined at the start of treatment versus upon completion of treatment were significant (p < 0.05), thus further demonstrating the effectiveness of chemotherapy for EOC (Figure 2/A-F, Table 3).

**Table 3:** Levels of CA-125, HE4, and 14-3-3 Z protein determined by ELISA and ECLIA methods

			**ELISA**			**ECLIA**		**ELISA**
**Patient no.**	**Chemo**	**CA-125**	**HE4**	**ROMA**	**CA-125**	**HE4**	**ROMA**	**14-3-3 Z**
	**Cyle no.**	**(U/mL)**	**(p/M)**	**%**	**(U/mL)**	**(p/M)**	**%**	**(ng/mL)**
*1*	1	23.38	95.87	26.19	105.70	79.63	46.90	38.28
	2	18.09	65.94	16.60	76.82	30.69	20.57	37.95
	3	7.78	64.47	9.49	73.55	15.77	11.20	18.78
	4	NA	NA	NA	NA	NA	NA	NA
	5	10.71	67.85	12.26	77.76	8.58	6.5	32.01
	6	7.90	62.81	9.35	75.21	9	6.60	39.82
*2*	1	26.93	334.49	58.99	353.50	176.10	83	0
	2	24.28	102.01	28	116.10	106.20	56	0
	3	19.31	66.42	17.37	79.16	29.24	20.10	0
	4	10.16	87.74	14.97	88.92	18.22	14.30	0
	5	3.33	81.38	6.7	88.28	15.06	12	0
	6	0.08	85.94	0.49	87.33	13.11	10.50	0
*3*	1	1.45	43.73	2	83.1	52.97	32.6	0
	2	0	49.24	0	67.49	28.04	17.70	0
	3	3.47	52.41	4.48	69.24	23.49	15.4	0
	4	NA	NA	NA	NA	NA	NA	NA
	5	0	44.09	0	52.38	17.25	9.7	0
	6	9.12	52.37	8.68	62.76	24.63	15.10	0
*4*	1	0	48.51	0	55.06	6.78	4	1.34
	2	0	55.9	0	58.43	6.91	4.30	1.42
	3	3.63	47.48	4.18	54.72	6.71	4	0.39
	4	2.55	45.86	3.15	53.12	6.08	3.50	0.32
	5	4.35	51.67	5.16	58.04	6.59	4.10	0.26
	6	NA	NA	NA	NA	NA	NA	NA
*5*	1	9.23	101.40	15.93	133.4	14.60	15.20	0
	2	12.40	93.07	17.77	107.40	8.69	8.20	0
	3	2.78	85.49	6.21	103.30	7.08	6.50	0
	4	14.24	69.76	15.03	75.26	6.66	4.90	0
	5	9.10	75.68	12.20	80.91	7.95	6.20	0
	6	9.73	74.47	12.55	80.21	7.89	6.10	0
*6*	1	153.04	904	93.53	1500	701.5	98.30	0
	2	445.65	904	96.94	1218	644.20	97.90	0
	3	368.55	714.78	95.57	843.10	394.10	95.50	0
	4	249.51	541.89	92.41	540.10	243.90	90.30	0
	5	95.43	259.90	73.68	227.30	84.13	65.40	0
	6	39.94	167.57	48.43	142	46.16	38.30	0
*7*	1	77.86	118.41	51.49	111.90	68.66	44.10	4.36
	2	17.01	122.23	26.50	124.90	22.64	22.64	15.02
	3	9.25	120.03	18.50	127	18.20	17.80	8.28
	4	8	107.76	15.47	111.10	16.34	15	6.38
	5	1.07	53.14	1.98	132.50	15.39	15.80	13.13
	6	9.03	202.84	27.82	186.80	26.99	30.30	5.74
*8*	1	475.23	259.74	90.07	248.50	4810	99.2	0.04
	2	500	90.83	75.92	80.07	1265	92.7	0
	3	189.18	67.99	53.34	70.60	642.10	85.20	0
	4	500	904	97.18	1500	5000	99.8	0
	5	172.63	57.11	47.22	57.21	254.10	65.30	0
	6	402.03	62.74	64.64	57.66	256.20	65.60	0
*9*	1	21.16	904	77.35	1500	185.40	93.70	2.46
	2	29.53	170.69	43.41	176.10	61.61	49.50	1.61
	3	4.97	97.08	10.38	103.70	21.29	18.10	1.32
	4	8.13	96.13	14.09	102.70	15.37	13.50	0
	5	5.90	105.57	12.56	108.90	13.93	12.80	0.13
	6	8.57	84.25	12.96	87.24	15.70	12.40	0.06
*10*	1	500	904	97.18	1500	5000	99.8	0
	2	189.91	62.04	51.08	60.85	243.10	65.30	0
	3	500	441.44	94.22	514.20	4789	99.5	0
	4	500	149.23	84.07	155.70	833.50	93.10	0
	5	84	127.48	54.97	121.70	197.90	71.60	0
	6	29.43	90.79	28.37	92.78	54.27	35	0
*11*	1	7.85	904	62.12	1500	117.70	90.20	0
	2	9.58	201.95	28.59	200.90	36.28	38.40	0
	3	0.93	131.04	4.42	125.90	18.88	18.30	0
	4	3.75	105.53	9.34	104.40	13.74	12.30	0
	5	2.65	107.64	7.54	107.80	13.41	12.30	0
	6	NA	NA	NA	NA	NA	NA	NA
*12*	1	126.02	397.04	84.2	404.30	1772	98.30	0.38
	2	500	365.72	93.07	323.20	1517	97.70	0
	3	192.79	273.67	83.2	258.90	761.40	94.70	0.09
	4	178.79	223.37	79.09	192.20	360.10	86.80	0
	5	121.86	168.58	68.17	169	265.70	81.30	0
	6	NA	NA	NA	NA	NA	NA	NA
*13*	1	500	904	97.18	1500	5000	99.8	0
	2	NA	NA	NA	NA	NA	NA	NA
	3	500	904	97.18	1500	5000	99.8	0
	4	500	904	97.18	853.90	3241	99.50	0
	5	120.65	355.03	82.16	349.30	171.90	82.5	0
	6	NA	NA	NA	NA	NA	NA	NA

ROMA, risk of malignancy algorithm.

NA, not available - when serum samples were too hemolyzed and measurements could not be made.

For 14-3-3 zeta protein levels detected in patients prior to chemotherapy by ELISA, the mean concentration was 1.93 ± 0.57 ng/ml. In contrast, the mean concentration of 14-3-3 zeta protein for healthy postmenopausal women (mean age, 58 y), was 0.39 ± 0.11 ng/ml. Subsequently, at the start of chemotherapy, the mean serum level of 14-3-3 zeta protein in EOC patients was 2.38 ± 1.44 pg/ml, and 2.17 ± 1.71 pg/ml after the final treatment. Neither the difference in levels detected for EOC patients and healthy postmenopausal patients, nor at the beginning and end of chemotherapy, was found to be significant (Figure 2/G).

### Correlation between radiological findings and serum parameters

**Figure 3:**
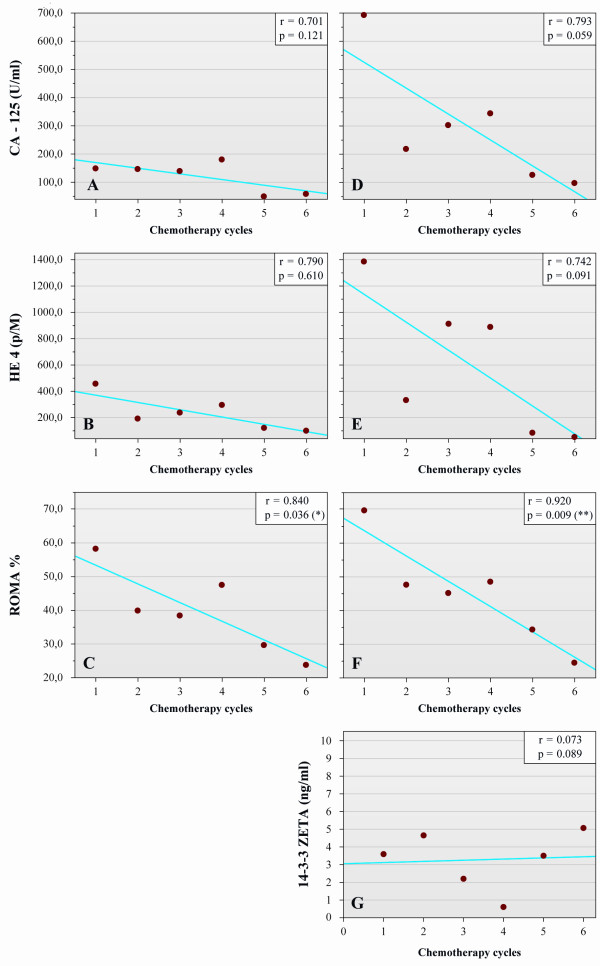
**Changes in CA-125, HE4, and 14-3-3 zeta protein serum levels, and ROMA index values during the six cycles of paclitaxel/carboplatin-based chemotherapy that were performed.** Mean concentrations of CA-125 determined by ELISA **(A)** and by ECLIA **(D)** are shown for each of the treatment days. Mean levels of HE4 determined by ELISA **(B)** and ECLIA **(E)** are also shown for each of the treatment days. Mean concentrations of 14-3-3 zeta protein were determined by ELISA **(G)** are represented at each chemotherapy days. Postmenopausal ROMA index values were calculated based on ELISA **(C)** and ECLIA **(F)** data. Linear regression analysis was performed for each diagram (see r value), and statistical significance was set at *p* < 0.05.

Neither ELISA nor ECLIA measurements of CA-125 and HE4 serum biomarkers provided significant linear regression correlations. However, the ROMA index values that were calculated based on these values did provide a strong significant regression correlation (r = 0.840, p = 0.036 and r = 0.920, p = 0.009, respectively) (Figure 3/C and F). Moreover, with a p-value margin of 0.01, a significant linear correlation was found for all ECLIA measurements. Linear regression analysis of 14-3-3 zeta protein levels at each treatment day, showed no significant correlation between the mean serum values and the chemotherapy cycles (r = 0.073; p = 0.089) (Figure 3/G).

Spearman’s correlation analysis further identified a significant correlation between CA-125 serum levels determined by ELISA and the largest tumor diameter measured by CT scans obtained following chemotherapy (p = 0.011). Levels of HE4 detected by ECLIA were also found to significantly correlate with tumor diameter (p = 0.04), while levels of 14-3-3 zeta protein did not significantly correlate with any of the examined parameters.

## Discussion

Several studies have demonstrated the limitations associated with depending on any single tumor marker for the detection of EOC. Initially, CA-125 was widely used. However, other malignant and benign diseases also express CA-125, thereby limiting its reliability as a tumor marker. In particular, CA-125 has a high false-positive rate among women with benign gynecological conditions such as endometriosis [14], and a low sensitivity in identifying patients with early-stage ovarian cancer [15]. Accordingly, when EOC is diagnosed, 80% of cases are in an advanced stage of disease (e.g., FIGO III-IV) [16]. To improve the specificity and sensitivity of an ovarian cancer diagnosis, additional tumor markers have been investigated. One novel tumor marker is HE4, which contains two whey acid protein (WAP) domains and eight cysteine residues that constitute a four-disulphide bond core [17]. HE4 localizes to human chromosome 20q12-13.1 and its expression significantly increases during malignant transformation. However, HE4 is expressed in normal tissues as well, and therefore, is not tumor specific. Correspondingly, it has been hypothesized that the function of HE4 is related to both spermiotelcosis (a protease inhibitor involved in sperm maturation) and natural immunity, although the mechanistic details of HE4 functions remain to be clarified [18]. As a tumor marker for the early detection of ovarian cancer, Moore et al. reported a sensitivity of 72.9% and a specificity of 95% for HE4 [7]. Moreover, when both HE4 and CA-125 were detected, the sensitivity increased to 76.4%. Therefore, the detection of more than one biomarker resulted in a 33.1% increase in the sensitivity of CA-125, and a 3.5% increase in HE4 sensitivity [7].

In the present study, ROMA values provided a PI based on the pre- or postmenopausal status of a patient, and the presence and levels of biomarkers CA-125 and HE4. As such, this PI relies on an accurate determination of serum levels of HE4 and CA-125. Moreover, in a recent study, the ROMA was found to be more effective in predicting ovarian cancer than the widely used risk of malignancy index (RMI), which employs ultrasound findings, CA-125 concentrations, and menopausal status [19]. Furthermore, when the specificity was set to 75%, the RMI had a sensitivity of 84.6%. For the same specificity, the sensitivity of the ROMA was significantly higher (94.3%). Although biomarker concentrations can be assayed by various methods (e.g., ELISA, chemiluminescent microparticle immunoassay), a recent study conducted by Ruggeri et al. demonstrated that chemiluminescent immunoassays are more adequate and more reproducible than commercially available ELISA kits that are characterized by interassay imprecision percentages (CV%) ranging from 6.8-10.3%, compared to < 4% for ECLIA [20]. The results of the present study are consistent with these findings, and they further support the use of the ECLIA method for routine determinations of CA-125 and HE4 levels. Furthermore, the deviation in accuracy for ELISA versus ECLIA can be attributed to the fully automated format of ECLIA, while ELISAs are manual assays that also require testing samples in duplicate.

14-3-3 zeta protein plays an important role in several different biological mechanisms. For example, it has been reported to be an adaptor protein for intracellular signaling since it contains tandem repeats of phosphoserine motifs that have the capacity to bind upstream and downstream signaling molecules [21-24]. 14-3-3 zeta protein also facilitate cell migration by forming a ternary complex with integrin alpha-4 and paxillin [23]. However, 14-3-3 zeta also has potential roles in cancerogenesis, based on its ability to bind NF-kappa B, beta-catenin, and Bcl-2, and to augment cancer cell proliferation [25]. Furthermore, 14-3-3 zeta protein has been shown to block activation of p38 mitogen-activated protein kinase (MAPK), thereby mediating an anti-apoptotic mechanism [26]. Numerous investigations have also suggested that 14-3-3 zeta protein is a key molecule in the malignant pathological processes of several malignancies, including oral, esophageal, lung, and breast cancers, as well as B cell lymphoma. Recently, He et al. reported that 14-3-3 zeta protein represents a candidate biomarker and a metastasis-promoting factor in ovarian cancer based on a serum proteomic analysis of a nude mouse xenograft model containing SKOV-3 cells and a mass spectrometry [liquid chromatography-tandem mass spectrometry (LC-MS/MS)] analysis to identify metastasis-related serum proteins [10]. Significantly higher expression of 14-3-3 zeta was detected in EOC patients than in patients with benign gynecological diseases. Furthermore, compared to CA-125, serum levels of 14-3-3 zeta protein was significantly upregulated when microscopic peritoneal metastasis was present, or when bilateral ovaries were involved. Accordingly, the authors suggested that 14-3-3 zeta protein may be a useful tool in differentiating FIGO stage Ib and Ic ovarian cancers from stage Ia ovarian cancers in the clinic [10]. However, the results of the present study are not consistent with these findings. For example, significant differences in the serum levels of 14-3-3 zeta protein was not detected in healthy menopausal women versus patients with advanced stage EOC. Furthermore, significant changes in serum levels of 14-3-3 zeta protein was not detected during the six consecutive cycles of chemotherapy treatment that were administered (Figure 2/G), although CT scans and CA-125 and HE4 levels unambiguously indicated the efficacy of the treatment. A possible explanation for these results is the insufficient number of patients enrolled in the current study. Thus, future studies should include a larger cohort in order to identify statistically significant changes. It is also possible that serum proteins may undergo degradation, even when stored at −80°C. In particular, it may be that 14-3-3 zeta is an unstable protein that needs to be assayed shortly after collection. Furthermore, an intriguing possibility is that 14-3-3 zeta may bind proteins activated by chemotherapeutic agents, or present as a result of chemotherapy, thereby obscuring detection of 14-3-3 zeta protein in serum. In the future s large-scale clinical investigation is necessary to evaluate the efficacy of 14-3-3 zeta protein, and to determine the sensitivity and the specificity of this biomarker comparing it to CA-125 and to HE4.

In conclusion, determination of CA-125 and HE4 serum levels for the ROMA represents a useful tool for the prediction of chemotherapy efficacy for EOC patients. However, based on our current findings, levels of 14-3-3 zeta protein were not found to reliably correlate with the clinical behavior of EOC, and therefore we question if it would be a useful biomarker for this disease.

## Abbreviations

EOC: Epithelial ovarian cancer; FIGO: International federation of obstetrics and gynecology; HE4: Human epididymis protein 4; ROMA: Risk of ovarian malignancy algorithm; CT: Computed tomography; LD: Longest diameter; ELISA: Enzyme-linked immunosorbent assay; ECLIA: Quantitative electrochemiluminescence assay; PI: Predictive index; SEM: Standard error of the mean; RMI: Risk of malignancy index; WAP: Whey acid protein; MAPK: Mitogen-activated protein kinase; LC-MS/MS: Liquid chromatography-tandem mass spectrometry.

## Competing interests

The authors have no conflict of interest with the present study to report.

## Authors’ contributions

IH has made substantial contributions to the conception and the design of this study, he also contributed significantly by collecting ovarian cancer samples. Furthermore he is responsible for all corrections and finalizations made to the manuscript.PG participated in the study design and coordination.TK along with LS and BP performed all the necessary immunoassays and biomarker measurements. AK performed the radiological assesments of all patients and is responsible for the interpretation within the manuscript of the radiological findings.NF performed all the statistical analysis necessary. BF participated in the design of the study, collection of samples and in addition,he has also been involved in drafting the manuscript. All authors read and approved the final manuscript and have given final approval of the version to be published.
